# Longitudinal observations of expected and actual library resource usage and barriers experienced by public health students

**DOI:** 10.5195/jmla.2020.691

**Published:** 2020-10-01

**Authors:** John Bourgeois

**Affiliations:** 1 jbou12@lsuhsc.edu, Ische Library, Louisiana State University (LSU) Health–New Orleans

## Abstract

**Objective:**

This longitudinal observational study explored relationships between actual and expected usage of library resources as well as anticipated and encountered barriers to that usage among public health affiliates over the course of a semester.

**Methods:**

School of Public Health master's degree students were sent questionnaires monthly throughout a semester that asked about usage of and barriers to library resources to examine changes over time.

**Results:**

Most students utilized library resources less often than they predicted at the beginning of the semester and did not have accurate expectations about which library resources they would use. Although most students encountered no difficulties using library resources, those who did often had multiple problems and seldom sought library assistance.

**Conclusion:**

As School of Public Health master's students had unrealistically high expectations of library resource usage, librarians may need to manage students' expectations and assist in overcoming difficulties. Further studies across health sciences disciplines are needed to determine differences between different populations of users.

## INTRODUCTION

Libraries seldom meet the needs of all users, often due to perceived barriers to library usage rather than active denial of service [1]. For example, recent studies of the nonuse of hospital libraries by health care professionals report barriers such as the perceived absence of a need for library services, lack of awareness of library services and resources, psychological factors such as lack of approachability, and time constraints [2, 3].

Limited literature has focused on public health user information needs and difficulties. A recent review analyzed the information needs of public health workers in terms of internal and external barriers to finding, accessing, and using information in their work [4]. This review revealed that political agendas lead to deprioritizing evidence-based decision-making, which resulted in information barriers, but the review did not directly examine public health workers' relationships with their libraries. Another study examined the information needs of public health students and concluded that librarians must offer continuous and targeted health resources for effective library marketing [5].

Although the definition of information needs can vary among settings [6], neither the relationships between library resources and public health users nor the barriers to accessing these resources have been the focus of previous research. Taking a broader view, research that examined barriers to using library resources at health sciences libraries found that the most common difficulties were related to time constraints and accessing resources [7, 8]. Yet, these observations were made at a single time point without any follow-up to investigate how perceived barriers may change over time.

Louisiana State University Health–New Orleans Libraries serve 6 schools. One is the School of Public Health (SPH), which has 100 graduate students, approximately 75 faculty members, and hundreds of staff who conduct numerous research programs on topics such as cancer, lead, and maternal and child health. The school currently offers a master's of science degree, master's of public health degree, and doctor of philosophy degree and will soon offer a bachelor's degree in public health. The school is served by a liaison librarian, who is housed at the main library, and offers all traditional services as well as more technologically advanced information services. The library has 3 floors of collaborative space with varying conversational volume levels as well as a 24-hour study commons. It provides access to over 60,000 monographs, 8,500 journals, and 87 databases. With all of these resources available to library users, it is important to understand how they interact with resources and what barriers they encounter, because public health users have unique needs [9] due to their community engagement and disparate backgrounds [5, 6].

Library users often have preconceived ideas about what a library is, what resources are available, and how they can use those resources. However, no research has examined how those perceptions change over time as users interact with the library. This longitudinal observational study, conducted over the course of a semester, examined changes in the actual and expected usage of library resources as well as anticipated and encountered barriers to library usage among SPH users.

## METHODS

### Participants

This research was funded by a Speedy Startup Grant from the South Central Academic Medical Libraries Consortium and was approved by the Institutional Review Board of Louisiana State University Health Sciences Center–New Orleans prior to participant recruitment and data collection.

This study was conducted during the fall 2017 semester. Eligible participants—who consisted of SPH students, faculty, and staff—were recruited when they first registered with the library, which is required for the use of most library resources. Registration occurs in person or via email and involves completing an application and scanning a barcode on their institutional identification cards. Because there were only sixty SPH library registrations in the previous fall semester, no sampling was performed to maximize participation in the study. Participants were recruited for this study by a librarian with the assurance that refusal to participate in the study would not impact access to library services or resources. Informed consent was attained from all participants.

### Data collection

Multiple questionnaires were administered electronically to participants via SurveyMonkey. The baseline questionnaire asked participants why they registered and how they learned about registering with the library, which library resources they believed they would use most often, and what difficulties, if any, they expected to encounter (supplemental Appendix A). Participants' year of birth and SPH program were obtained for demographic purposes. Regardless of when in the fall 2017 semester a SPH user registered, they were offered the opportunity to participate in the study. The rationale was that those registering later in the semester might have different experiences and motivations than those registering at the beginning of the semester. Therefore, their inclusion was deemed beneficial for enriching the representativeness of the data.

Starting one month after completing the baseline questionnaire, participants were sent follow-up questionnaires to gather information on their usage of library resources in the previous month, including which library resources they used, how often, and for how long (supplemental Appendix B). The questionnaire also asked about barriers to library access, how participants overcame those barriers, predicted usage of resources in the coming month, and any expected difficulties. A maximum of two follow-up questionnaires were administered on a monthly basis prior to the final questionnaire. At the end of the semester, participants were sent a final questionnaire that asked them to recall their library use in the previous month, their reflections on the entire semester, and their predicted usage and barriers in the spring 2018 semester (supplemental Appendix C).

As this study utilized a longitudinal design to determine how participants' attitudes changed over time, loss at follow-up was a major issue. To mitigate this, participants received up to 2 reminder emails to complete each questionnaire at 1-week intervals. Following the completion of each questionnaire, participants were given a $5 gift card, which had to be picked up in-person at the library circulation desk. After the end of the semester, all identifiers were stripped from the data set, leaving only the last 4 digits of the user's barcode as a unique identifier.

### Analysis

All questionnaires allowed participants to enter additional information in free-text “other” fields. For example, participants could indicate other resources or barriers that they encountered or expected to encounter. The librarian did not look at the questionnaire data during the fall 2017 semester because doing so could have changed interactions with SPH affiliates, possibly introducing bias. Most completed “other” fields could be recoded into an existing field and included in the analyses. No “other” field responses were omitted.

Risk ratios (RRs) were calculated to evaluate whether participants' planned usage of resources predicted their actual usage. Data were analyzed using IBM SPSS 24.

## RESULTS

Thirty-eight SPH users registered with the library in fall 2017. Of these, 15 master's students completed the baseline questionnaire, the 2 follow-up questionnaires, and final questionnaire, resulting in an overall response rate of 39.5% (Table 1). Only these 15 users were included in the longitudinal analysis. Most users (80.0%, n=12) first heard about registering with the library at the SPH orientation, although some were encouraged to register by SPH coordinators (13.3%, n=2). Over half of users (60.0%, n=9) registered simply to complete the task.

**Table 1:** Response rates by questionnaire

	Baseline	Follow-up 1	Follow-up 2	Final	Overall
Invited	38	27	20	18	38
Completed	27	20	18	15	15
Response rate	71.1%	74.1%	90.0%	83.3%	39.5%

Across all library resources, expected usage was highest at the beginning of the semester and declined as the semester progressed. Generally, expected usage was higher than actual usage except for articles, journals, and databases in the third month of the study and print books and reserves over the entire semester. As the semester progressed, electronic resources (i.e., articles, journals, and databases; electronic books) showed the most severe decline in expected and actual usage, although expected and actual usage values appeared to converge by the third month. Quiet space was the most consistently used resource, although its expected usage was also higher than its actual usage.

**Figure 1:**
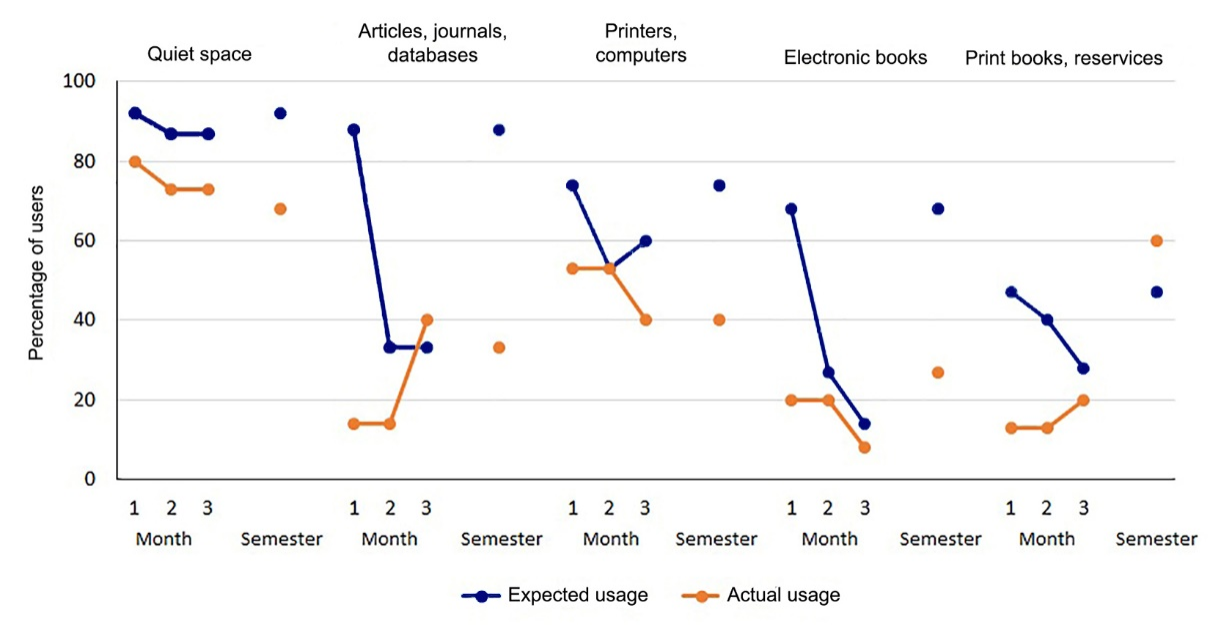
Expected and actual library resource usage across time

For some library resources, expected usage predicted actual usage (Figure 1). Users who indicated that they planned to use quiet space (RR: 3.50, 95% confidence interval [CI]: 1.53–8.01, *p*=0.003) or articles, journals, and databases (RR: 1.86, 95% CI: 1.12–3.07, *p*=0.016) at the beginning of the semester were significantly more likely to use these resources throughout that semester. Also, users who indicated that they planned to use quiet space (RR: 3.50, 95% CI: 1.53–8.01, *p*=0.003) or electronic books (RR: 1.67, 95% CI: 1.01–2.77, *p*=0.048) at the beginning of the semester were significantly more likely to report planning to use these resources during the following semester. Aside from these relationships, there were no other significant relationships between predicted resource use and other variables.

**Figure 2:**
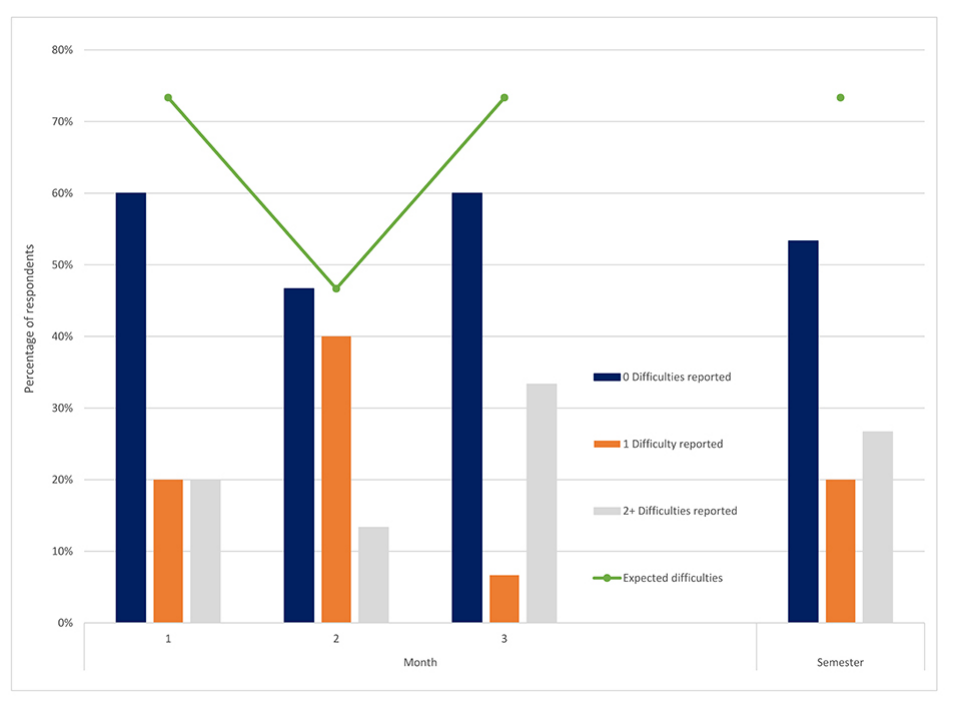
Expected and actual difficulties encountered over time

Overall, users encountered fewer difficulties than they expected (Figure 2) (Table 2). Although approximately 70% of users expected difficulties, less than 50% experienced difficulties during most questionnaire periods. Among those who reported experiencing difficulties, roughly half had multiple types of difficulties. The most frequently reported difficulty was “finding time,” although “not knowing how to use [library resources]” and “getting off-campus access” were other common difficulties.

**Table 2:** Overall frequency of reported difficulties

Reported difficulties	Count	Percentage
Finding time	39	50.6%
Not knowing how to use library resources	23	29.9%
Navigating the library's website	4	5.2%
Getting off-campus access	11	14.3%
Total	77	100.0%

Although expected difficulties remained high throughout the semester and some users experienced multiple difficulties, rarely did they attempt to contact the library to rectify these issues. When asked how they attempted to overcome the encountered difficulties, the most prevalent response was “I did not bother with it.” Although the frequency of this response lessened over the course of the semester, many users still did not often use the library's access points. Seeking assistance improved with time but still remained far below needed levels of assistance.

## DISCUSSION

Except for certain library resources, expected usage generally did not predict actual usage. Overall, users used library resources less and had fewer difficulties than anticipated. However, half of users who encountered difficulties tended to have multiple difficulties. Furthermore, users made few attempts to overcome these difficulties, although attempts to overcome barriers increased as the semester progressed.

### Outreach

Knowing what drives users to register for library access reinforces the importance of relationships that seem intuitive and now have empirical evidence. In this study, the key drivers of SPH user library registrations were attendance at the SPH orientation and encouragement by SPH coordinators, which confirmed the need to cultivate and maintain relationships with particular schools and their coordinators [10, 11], who organize orientation and serve active roles in enabling librarians to directly engage with users. Similarly, recruiting faculty to become involved in student library registration may promote more library engagement [12, 13].

### Library resource usage

Initially, SPH library users tended to have overly optimistic expectations about how often they would use library resources and did not accurately predict which resources they would use. As the semester progressed, however, their actual use became more closely aligned with their predictions. Of all library resources specified in the questionnaires, quiet space was consistently the resource with the most anticipated and actual usage. Some reasons for this finding might be that simply occupying space does not require any special knowledge, and quiet space is a familiar resource that is common across all types of libraries. Also, previous studies show that library users place a high value on physical library space and recommend improving libraries' spatial layouts and ambiance to engage present and future users [10, 14, 15]. In turn, library space can be promoted as an open, available meeting area.

Considering electronic resources (i.e., articles, journals, and databases; electronic books), users' expected usage was far greater than their actual usage. By the first follow-up questionnaire, however, students had realigned their expectations with actuality. This observation has at least two non-mutually exclusive interpretations. First, the library might not be thoroughly integrated into the SPH curriculum. Second, librarians might not need to emphasize these library resources during orientation if they are not frequently used.

### Barriers to library usage

Although SPH users appeared to become better at predicting their actual usage of library resources over time, it is unclear why more users expected difficulties than actually encountered barriers in using those resources. While difficulty finding time was the most commonly reported barrier, not knowing how to use library resources and accessing resources from off-campus were also barriers that users encountered, similar to findings in previous studies [7, 8, 10, 11].

Although no studies have explicitly examined why library users do not ask for help, a growing body of evidence offers potential explanations. In particular, individuals' nonuse of library resources has been associated with perfectionism [16]. That is, if users who exhibit perfectionistic characteristics have unrealistically high standards for themselves and base their self-worth on their productivity, they may disengage from available resources if accessing those resources initially proves difficult. A lack of self-confidence and fear of seeking assistance may also lead to the nonuse of library resources [10].

However, more SPH users reported asking for assistance as the semester progressed. One reason for this finding might be because users procrastinated until the end of the semester [10]. Alternatively, users might have become more familiar with the library over time, making it easier for them to seek assistance in using library resources [14].

### Limitations

Despite inviting all new SPH users to participate in this study, only master's students opted to participate, consistent with previous findings that faculty members are less likely to engage in library training [12]. However, as only four new SPH faculty members registered for the library (out of a total of thirty-eight new registrants), it was unlikely that their participation would have substantially altered the results.

Another observation of note arose from the final questionnaire, in which users were asked about their use of library resources over the past semester and past month. When responses regarding the past semester were compared against those from the follow-up and final questionnaires for validity, there were discrepancies in reported usage across all library resources. For example, some users reported that they used quiet space in the past semester but did not report such use in the previous monthly questionnaires. Such discrepancies were randomly distributed among users and resources, and no attempts were made to resolve them in the present analysis. However, this phenomenon is important to note when designing future studies.

### Recommendations

The results of this study support two potential initiatives for addressing the problems that were uncovered [17]. First, managing expectations early, perhaps during orientation, would align students' planned resource usage with their actual usage and allow them to obtain a better sense of the time necessary for utilization of library resources and to develop time-management skills for their graduate programs. Indeed, the most frequently reported barrier to library usage was difficulty finding time. Explicitly acknowledging this problem during orientation could help users better plan their time [14, 18].

Second, library users should be engaged in a setting with which they are familiar or comfortable. This may mean engaging users in their schools instead of in the library. In the past, the only opportunity that the librarian had to interact with SPH users was orientation, which might have resulted in a disconnect between the school and the library. Since then, the librarian has taken steps to become more integrated into the SPH, including getting on the school's mailing list, having office hours in the school, and attending the school's social and professional events. Because the library only has a presence at student orientation, it may also be helpful to engage faculty during new employee orientation and to create library orientation videos [19].

As this study focused on users from one public health school during a single semester, the results cannot be generalized to a broader population of library users, especially considering that students from different disciplines use library resources differently [20]. Therefore, future studies could be conducted across one or more multidisciplinary health sciences centers for a full year to better understand library resource usage and barriers.

## SUPPORTING AGENCY

South Central Academic Medical Libraries Consortium (SCAMeL).

## SUPPLEMENTAL FILES

**Appendix A:** Baseline survey

**Appendix B:** Monthly follow-up survey

**Appendix C:** Final survey
