# Supplementary material for: Longitudinal observations of expected and actual library resource usage and barriers experienced by public health students
Source: J Med Libr Assoc. 2020 Oct 1;108(4):618–24. doi: 10.5195/jmla.2020.691 (PMC7524613; doi:10.5195/jmla.2020.691)
Supplement: Supplementary file 1 — Appendix A: Baseline survey [file jmla-108-4-618-s01.pdf]

JMLA Journal of the Medical Library Association

SC

Supplemental content to
J Med Libr Assoc. Oct;108(4):dx.doi.org/10.5195/jmla.2020.691
www.jmla.mlanet.org
© Bourgeois 2020

Longitudinal observations of expected and actual library resource usage and barriers experienced by public health students

John Bourgeois, AHIP

APPENDIX A

Baseline survey

1. Where did you **first** hear about registering with the library?
a. School of Public Health (SPH) orientation
b. Library lecture during class
c. Recommendation from peers
d. Visiting the library
e. SPH coordinators
f. Resource Fair
g. Peers
h. Other. Please specify: ______________________________

2. What eventually **led** you to register with the library?
a. Wanted to get it taken care of, just in case
b. Needed articles for classes
c. Needed books/reserves for classes
d. Wanted off-campus access
e. Needed to find articles for professional research
f. Opportunity to participate in this research
g. Had the registration form right in front of me at a fair, lecture, etc.
h. Other. Please specify: ______________________________

3. What library resources do you think you will use this semester? Select all that apply.
a. The quiet space
b. Articles/journals/databases
c. Electronic books
d. Print books/reserves
e. Printers/computers
f. Unsure what resources are available
g. None
h. Other. Please specify: ______________________________

JMLA Journal of the Medical Library Association

SC

Supplemental content to
J Med Libr Assoc. Oct;108(4):dx.doi.org/10.5195/jmla.2020.691
www.jmla.mlanet.org
© Bourgeois 2020

4. Of the library resources you indicated in question 3, how often do you think you will use them?

Resource	Daily	Several times a week	Weekly	Several times a month	Once a month	Several times a semester	Once a semester	Never/not applicable
Quiet space								
Articles/ journals/ databases								
Electronic books								
Print books/ reserves								
Printers/ computers								
Other								

5. What difficulties do you anticipate to using the library resources? Select all that apply.
a. Finding time
b. Not knowing how to use them
c. Navigating the library's website
d. I don't anticipate facing any problems
e. I anticipate problems but do not know specifically what
f. Other. Please specify: ____________________

6. How long have you been at Louisiana State University Health–New Orleans (LSUHSC–NO)?
a. ≤ 6 months
b. 6 months but ≤ 1 year
c. 1 year but ≤ 2 years
d. 2 year but ≤ 3 years
e. 3 years

7. What department in the SPH are you associated with?
a. Behavioral and Community Health Sciences
b. Biostatistics
c. Environmental and Occupational Health Sciences
d. Epidemiology
e. Health Policy and Systems Management
f. Not applicable. Not in a listed department

8. In what year were you born? ____________________

9. What are the last 4 digits of your library barcode: ____________________

10. Please include any comments that you feel would be beneficial for this study.
