# Supplementary material for: Longitudinal observations of expected and actual library resource usage and barriers experienced by public health students
Source: J Med Libr Assoc. 2020 Oct 1;108(4):618–24. doi: 10.5195/jmla.2020.691 (PMC7524613; doi:10.5195/jmla.2020.691)
Supplement: Supplementary file 3 — Appendix C: Final survey [file jmla-108-4-618-s03.pdf]

JMLA Journal of the Medical Library Association

SC
Supplemental content to
J Med Libr Assoc. Oct;108(4):dx.doi.org/10.5195/jmla.2020.691
www.jmla.mlanet.org
© Bourgeois 2020

Longitudinal observations of expected and actual library resource usage and barriers experienced by public health students

John Bourgeois, AHIP

APPENDIX C

Final survey

In the past month

1. What library resources have you used in the past month? Select all that apply.
a. The quiet space
b. Articles/journals/databases
c. Electronic books
d. Print books/reserves
e. Printers/computers
f. None
g. Other. Please specify: ______________________________

2. Of the resources you indicated in question 2, how often have you used them in the past month?

Resource	Daily	Several times a week	Weekly	Several times a month	Once a month	Never/not applicable
Quiet space						
Articles/journals/databases						
Electronic books						
Print books/reserves						
Printers/computers						
Other resources						

JMLA Journal of the Medical Library Association

SC
Supplemental content to
J Med Libr Assoc. Oct;108(4):dx.doi.org/10.5195/jmla.2020.691
www.jmla.mlanet.org
© Bourgeois 2020

3. In the past month, what is the overall time you spent using the each of the library's resources?

Resource	≤ 1 hour	1–4 hours	4–8 hours	8–12 hours	12–16 hours	16–20 hours	20–24 hours	> 24 hours	Never/not applicable
Quiet space									
Articles/journals/ databases									
Electronic books									
Print books/ reserves									
Printers/computers									
Other resources									

4. In the past month, what library resources would you have liked to use but for whatever reason were unable to? Select all that apply.
a. The quiet space
b. Articles/journals/databases
c. Electronic books
d. Print books/reserves
e. Printers/computers
f. None. Could use all desired resources
g. Other. Please specify: ____________________

5. What problems have you had using these resources?

Resource	Finding time	Not knowing how to use it	Navigating the library's website	Getting off-campus access	Other difficulty	Not applicable
Quiet space						
Articles/journals/ databases						
Electronic books						
Print books/reserves						
Printers/computers						
Other resources						

6. If relevant, please elaborate on any other difficulties you've had using the library resources in the past month.

____

JMLA Journal of the Medical Library Association
SC
Supplemental content to
J Med Libr Assoc. Oct;108(4):dx.doi.org/10.5195/jmla.2020.691
www.jmla.mlanet.org
© Bourgeois 2020

7. How have you tried to overcome these problems?
a. Calling the library
b. Emailing the library
c. Using the chat box on the library's home page
d. Coming to the library in person
e. Did not bother with it
f. Not applicable. Had no problems
g. Other. Please specify: ______________________________

In the past semester

8. Since registering with the library, what resources do have you used? Select all that apply.
a. The quiet space
b. Articles/journals/databases
c. Electronic books
d. Print books/reserves
e. Printers/computers
f. Don't know
g. None
h. Other. Please specify: ______________________________

9. Of the library resources you indicated in question 7, how often have you used them?

Resource	Daily	Several times a week	Weekly	Several times a month	Once a month	Several times a semester	Once a semester	Never/not applicable
Quiet space								
Articles/ journals/ databases								
Electronic books								
Print books/ reserves								
Printers/ computers								
Other resources								

JMLA Journal of the Medical Library Association

SC

Supplemental content to
J Med Libr Assoc. Oct;108(4):dx.doi.org/10.5195/jmla.2020.691
www.jmla.mlanet.org
© Bourgeois 2020

10. In the past semester, what library resources would you have liked to use but for whatever reason were unable to? Select all that apply.
a. The quiet space
b. Articles/journals/databases
c. Electronic books
d. Print books/reserves
e. Printers/computers
f. Not applicable. Could use all desired resources
g. Other. Please specify: ______________________

11. What have you found to be the biggest difficulties to using the library resources?

Resource	Finding time	Not knowing how to use it	Navigating the library's website	Getting off-campus access	Other difficulty	Not applicable
Quiet space						
Articles/journals/ databases						
Electronic books						
Print books/reserves						
Printers/computers						
Other resources						

12. If relevant, please elaborate on any other difficulties you've had using the library resources in the past semester.

____

In the next semester

13. What library resources do you think you will use next semester?
a. The quiet space
b. Articles/journals/databases
c. Electronic books
d. Print books/reserves
e. Printers/computers
f. Don't know
g. None
h. Other. Please specify: ______________________

JMLA Journal of the Medical Library Association

SC

Supplemental content to
J Med Libr Assoc. Oct;108(4):dx.doi.org/10.5195/jmla.2020.691
www.jmla.mlanet.org
© Bourgeois 2020

14. Of the library resources you indicated in question 13, how often do you think you will use them next semester?

Resource	Daily	Several times a week	Weekly	Several times a month	Once a month	Several times a semester	Once a semester	Never/not applicable
Quiet space								
Articles/ journals/ databases								
Electronic books								
Print books/ reserves								
Printers/ computers								
Other resources								

15. What do you anticipate being the biggest difficulties to using the library resources next semester? Select all that apply.
a. Finding time
b. Not knowing how to use it
c. Navigating the library's website
d. I don't anticipate facing any problems
e. I anticipate problems but do not know specifically what
f. Other. Please specify: ______________________________

Participant information

16. What department in the School of Public Health are you associated with?
a. Behavioral and Community Health Sciences
b. Biostatistics
c. Environmental and Occupational Health Sciences
d. Epidemiology
e. Health Policy and Systems Management
f. Not applicable. Not in a listed department.

17. Please enter the last 4 digits of your library barcode: ______________________

18. Please include any comments that you feel would be beneficial for this study.
